# Letter to the Editor: “Cost‐effectiveness and budget impact analysis of the implementation of differentiated service delivery models for HIV treatment in Mozambique: a modelling study”: Resource reductions are not equal to cost savings

**DOI:** 10.1002/jia2.26367

**Published:** 2024-10-15

**Authors:** Sydney Rosen, Nkgomeleng Lekodeba, Linda Sande, Brooke Nichols

**Affiliations:** ^1^ Health Economics and Epidemiology Research Office Faculty of Health Sciences University of the Witwatersrand Johannesburg South Africa; ^2^ Department of Global Health Boston University School of Public Health Boston Massachusetts USA; ^3^ Department of Global Health Amsterdam Institute for Global Health and Development Amsterdam UMC University of Amsterdam Amsterdam Netherlands; ^4^ FIND Geneva Switzerland

1

For the past decade, differentiated service delivery has been a major focus of national HIV treatment programmes in sub‐Saharan Africa [1, 2, 3]. While its main objective has been to make antiretroviral therapy (ART) provision more client‐centred, it has also been seen as a way to increase the efficiency of ART delivery, largely by lowering the “intensity” of care by allowing less frequent clinic visits, longer medication dispensing intervals, out‐of‐facility service locations, and, in some cases, task shifting to lower‐paid or less skilled staff cadres [4, 5]. The few published studies of the costs of differentiated service delivery (DSD) models have had conflicting results, with the simplest models of care, such as facility‐based 6‐month dispensing of medications, appearing to cost less than conventional care per client served and other models, such as adherence clubs, potentially costing more [6, 7].

Given the variation in cost results to date, we read with interest the paper by Uetela et al. [8] reporting their cost‐effectiveness and budget impact analysis of DSD models for HIV treatment in Mozambique. Studies of this type turn out to be far more challenging to conduct than they first appear, because of the difficulty of defining a comparison population, obtaining complete individual‐level data on resource utilization, observing actual resource utilization for health system interactions that often occur outside fixed healthcare facilities, accounting for participants who switch models during the study observation period, and incorporating individual facility idiosyncrasies in model implementation. We therefore congratulate the authors for their effort in pulling together all the disparate types of data needed to make these estimates.

We do, however, have one major concern about this paper's conclusions that we believe should be called to readers’ attention. The paper states that “the implementation of these models will result in savings of approximately US$14 million to the health system between 2022 and 2024.” It is critical to note that, as far as we can tell, none of these “savings” is in fact a cash or budgetary saving to the health system. The “savings” reported are generated primarily by a reduction in the use of healthcare provider time required by the DSD models. This is time that facility managers can reallocate to other purposes, and it may allow them to see more clients or provide higher‐quality care to existing clients, but it does not represent money saved, unless absolute numbers of healthcare staff are reduced, for example by laying off nurses or pharmacy technicians. We have never encountered a healthcare system in this region that is either able or willing to reduce its total complement of healthcare workers in response to the advent of DSD models. No mechanism or pathway exists for DSD models to “save money.” They do, without doubt, save resources (e.g. staff time, facility space), and it is desirable and likely that these resources can be used to produce more health for other clinic clients. DSD will not, however, reduce the Ministry of Health's ART budget. Policymakers should take this into account in considering the net benefits of DSD.

## COMPETING INTERESTS

The authors report no competing interests.

## AUTHORS’ CONTRIBUTIONS

SR: Original draft of the manuscript. All authors contributed to the overall message of the manuscript, revised the draft, and reviewed and approved the final manuscript.

## FUNDING

Funding for the study was provided by the Bill & Melinda Gates Foundation through INV‐037138 to the Wits Health Consortium and INV‐031690 to Boston University. The funders had no role in study design, data collection, analysis, or interpretation of data or in the writing of this manuscript.

## Data Availability

No data were utilized in this manuscript.
